# Factors Affecting the Sexual Behavior of Korean University Students

**DOI:** 10.3390/healthcare11131837

**Published:** 2023-06-23

**Authors:** Mi-Kyoung Cho, Mi Young Kim

**Affiliations:** 1 Department of Nursing Science, Chungbuk National University, 1 Chungdae-ro, Seowon-gu, Cheongju KR28644, Republic of Korea; 2 College of Nursing, Hanyang University, 222 Wangsimni-ro, Seongdong-gu, Seoul KR04763, Republic of Korea

**Keywords:** sexual attitude, sexual behavior, pornographic video, university student

## Abstract

Recent incidents in Room n, sexual harassment by social leaders, and the #MeToo movement showed Korea’s immature and distorted sexual culture. This cross-sectional descriptive study investigated the factors affecting the sexual behavior of Korean university students. The participants comprised 258 university students from S and C. The data collection period was from 29 November 2021 to 3 December 2021, and an online survey was conducted on sexual behavior, sexual attitudes, and subject characteristics. The collected data were analyzed using PASW Statistics 25.0. The average age of the participants was 21.38 ± 1.62 years old; the average age when they first watched a pornographic video on YouTube was 14.25 ± 2.55 years old. Sexual behavior was statistically significantly higher for men over 21 and under 14 when they first watched a pornographic video. As the age of the subjects increased, the younger the age of viewing pornographic videos and the thumbnail viewing path of the pornographic videos affected sexual behavior, with an explanatory power of 11.0% (F = 6.27, *p* < 0.001). Higher sexual attitudes in the communion and permissiveness domains showed greater influence on sexual behavior; the explanatory power was 24.0% (F = 10.02, *p* < 0.001). Korean university students must be educated on sex early to develop correct sexual attitudes and engage in correct and responsible sexual behaviors in their youth.

## 1. Introduction

South Korea has experienced social controversies over sexual issues and crimes, including the Nth Room case, incidents of sexual harassment by leaders of society, and the “Me Too” movement [1]. With its rapid industrialization, economic growth, and the proliferation of mass media, Korean society has shifted its focus from traditional Confucian values to modern values and the trend of sexual openness. Meanwhile, as an increasing number of adolescents and college students are being exposed to pornography in the media and on the smartphone, their sexual consciousness and attitudes are becoming more open and liberal [2].

College students are particularly likely to translate their sexual knowledge into sexual behaviors as they are no longer under their parent’s control, and they begin to extend the scope of their relationship with friends and start a romantic relationship in some cases [3]. The level of openness regarding sexual attitudes and experiences has increased [4]. Moreover, many college students are exposed to commercialized sexual content in various media. This triggers sexual arousal, leading to incorrect sexual knowledge. Under these circumstances, their attitudes and knowledge of sex may become distorted [5]. Based on a survey conducted in South Korea in 2021, findings reveal that 3.5 percent of female respondents reported instances of dating violence [6]. College students may be exposed to negative situations such as dating violence and rape by imitating commercial footage or exaggerated situations.

This study focused on social media, but it was added that family, school, and peer relationships primarily influenced it. The sexual socialization of adolescents in Korea is affected by various environments. First, it is primarily a family and is influenced by school and peer relationships. Furthermore, college students are in early adulthood, and this life cycle is particularly important for building diverse and significant relationships. Therefore, it is essential to help them obtain sound values regarding sexual behaviors to nurture the next generation to become valuable members of society and prepare them for the future [7]. Sound values are about understanding and knowing the importance of sex and the right to self-determination without seeing sex commercially. As sex culture changes, Korean society will need to harmonize individual attitudes toward sex and attitudes required by society. However, if they obtain incorrect sexual knowledge through online pornography during such a significant period, they could face difficulties building sound and correct sexual attitudes and creating a healthy sex culture, as they could have distorted sexual views and experiences based on online pornography [8].

Sexual content is found widely across the media, not only in South Korea but also in many other countries. Aside from sexually explicit media content and pornography, sexual content containing verbal or visual expressions implying sexual relationships, courtship, or sexual intercourse accounts for over 80% of all movie and television content [9]. Additionally, exposure to sexual content and attitudes described in television, music videos, movies, video games, and song lyrics can affect the sexual attitudes of users [9]. In a study on the influence of pornography, among responses indicating a negative experience with pornography, identified themes included increased feelings of inadequacy, decreased sexual satisfaction in a relationship, a sense of unrealistic expectations, and feelings of betrayal. However, there were also positive effects; the emergent themes centered on increased diversity of sexual experiences, increased sexual gratification, increased satisfaction with a partner, and erotic climate in a relationship [10].

People experience and enjoy various types of content as they use more diverse media. YouTube is a video service platform that Internet users have been using for a long time. More than 90% of Internet users watch YouTube videos, and the rise in popularity of algorithm-based AI recommendation systems means that people watch content recommended by the algorithm [11]. This environment exposes people to media containing sexual content and affects the sexual attitudes and behaviors of college students.

Adolescents are constantly supervised to prevent exposure to sexually harmful content. However, since college students are regarded as adults and are therefore considered to have some degree of autonomy, people do not treat them similarly in this context. It is believed that college students are free to do anything regarding sexual behavior. However, there are several limitations to developing unbiased Sexual Knowledge, Sexual Attitudes, and Perceptions. Specifically, because things have changed much from the past when few people are exposed to pornography, it becomes necessary to investigate whether online pornography affects the sexual behaviors of college students.

Sexual attitudes refer to an individual’s opinions concerning sexual intercourse and psychology. As college students with positive sexual attitudes have been found to have a higher level of sexual autonomy [12], we need to examine the sexual attitudes of college students. Previous studies on the sexual attitudes of college students in South Korea have investigated whether they are conservative or liberal in their sexual attitudes. The results showed that 26.3% of the study participants had conservative sexual attitudes, while 73.7% had liberal sexual attitudes [13], indicating that most participants had liberal sexual attitudes. In particular, it has been reported that in South Korea, the number of college students who have had premarital sexual intercourse has rapidly increased as their sexual attitudes become more liberal, but most have not had a medical check-up concerning their sexual experience [14]. This situation shows that sexual experiences can raise issues regarding sexual health. Consequently, factors influencing the sexual behaviors of college students need to be explored to maintain sexual health in the recent trend of freely enjoying a sexual life with open-mindedness.

Considering the swiftly changing environment and values of college students, it is necessary to examine and analyze the sexual behaviors of college students, in addition to the influencing factors. In this regard, this study aimed to the factors through which sexual attitudes influence sexual behaviors in South Korean university students.

## 2. Methods

### 2.1. Study Design

This cross-sectional descriptive study aimed to identify the general characteristics of college students in South Korea, characteristics of online pornography, and factors through which sexual attitudes influence sexual behaviors.

### 2.2. Study Subjects

The subjects of this study were college students in S and C cities. Among these students, those who provided consent to participate in this study after reading an online explanation of the methods and purpose of the study completed an online survey. For subject selection, G*power version 3.1.9.6 software was used to calculate the sample size, with a medium effect size of (f2) = 0.10, a significance level of (α) = 0.05, power for testing of (1 − β) = 0.90, 7 tested predictors of multiple linear regression, and 11 predictors in total. Therefore, the sample size of 226 was calculated. Considering the dropout rate of approximately 10%, the target sample size was 251. At the end of the online survey period, 258 participants responded. All questionnaires collected were used for the final analysis.

### 2.3. Ethical Considerations

To protect the subjects of this study, relatively vulnerable students, only those who voluntarily agreed to participate were recruited in principle. The identity and contact information of the researcher was shared, and the study’s purpose, methods, and details were explained before the survey was conducted. Only those participants who provided consent to participate in the study answered the survey. The participants were also informed that the data collected through the survey would be processed anonymously to protect their identity and privacy, that the data would be used only for the study, and that they could withdraw from the study at any time.

### 2.4. Research Instruments

#### 2.4.1. Sexual Behavior

Sexual behavior is the behavior individuals exhibit to gratify one of their basic needs, namely, their sexual needs [15]. This study used the sexual behavior instrument developed by Kwon [16]. It comprises 12 items that cover the history of romantic relationships, handholding, kissing, caressing, watching pornographic videos, sexual impulses, masturbation, homosexuality, sexual intercourse, sexually transmitted diseases, pregnancy, and sexual violence. Each item is scored on a 4-point Likert scale (1 = “absolutely disagree” and 4 = “absolutely agree”). The sexual behavior tool consisted of the initial sexual experiences (behaviors 1–7 items) and the advanced sexual experiences (behaviors 8–12 items). The higher the mean score in each sub-domain, the greater the number of sexual experiences. Regarding the instrument’s reliability in Kwon’s [16] study, Cronbach’s α was 0.84, while it was 0.81.

#### 2.4.2. Sexual Attitudes

Sexual attitudes define values and beliefs about sexuality and are based on family and cultural views regarding sexuality, sex education (both formal and informal), and prior sexual experiences [17]. This study used an instrument translated and reverse-translated by a Korean and English bilingual person from the Brief Sexual Attitudes Scale (BSAS) by Hendrick et al. [18]. The BSAS comprises four subcategories: birth control (responsibility in birth control), communion (attitude toward the importance of bonding with a sexual partner), instrumentality (attitude toward enjoying physical sex), and permissiveness (permissiveness toward an open relationship). This instrument was translated into Korean after its face validity was confirmed by three nursing professors. The BSAS comprises 23 items: three on birth control, five on communion, five on instrumentality, and ten on permissiveness. Each item is measured on a 5-point Likert scale, ranging from one point for “strongly disagree” to five points for “strongly agree”. A higher score indicates a higher degree of acceptance of an individual’s sexual attitude as a social value in each subcategory. In the study by Hendrick et al. [18], Cronbach’s α for the subscales of birth control, communion, instrumentality, and permissiveness were 0.84, 0.71, 0.77, and 0.93, respectively, while it was 0.60, 0.79, 0.75, and 0.87, respectively.

#### 2.4.3. Participant Characteristics

Among the general characteristics of the participants, age, gender, and religion were included in the demographic characteristics, and the environment-related characteristics included age at first viewing online pornography, how to watch online pornography, reasons to watch online pornography, and YouTube viewing time for the last week.

### 2.5. Data Collection

This study collected data from November 29 to December 3, 2021. The researcher recruited study participants from ‘Everytime’, an online community for college students, and Kakao Talk open chat rooms. ‘Everytime’ is a student community of 397 campuses nationwide in South Korea. It is the most actively used student community at many universities through an anonymous system for safe conversation among students who have passed school certification and an online community platform where students open and operate bulletin boards. In ‘Everytime’, students can conveniently manage their studies by creating timetables, class schedules, and to-dos, accessing useful school life information such as learning, and using an anonymous community to communicate with students on the same campus. The researchers explained the purpose and method of the study to the student representative of their university. They asked them to fill out an online consent form and questionnaires at the online bulletin board of ‘Everytime’, and the open chatting room of KakaoTalk, through the online URL. The participants were provided with an online explanation regarding their consent for participation in the study, the rights of study participants, the protection of privacy of study participants, and the purpose of the study. Only those who voluntarily consented to participate in the study were selected, and they answered the survey.

### 2.6. Data Analysis

The collected data were analyzed using PASW Statistics 25.0 (Chicago, IL, USA). The characteristics of the participants were analyzed using frequency, percentage, mean, and standard deviation. Sexual behaviors and attitudes, the main variables of this study, were analyzed using the converted scores, standard deviation, and ranges. The differences in sexual behaviors (including early sexual activity and advanced sexual activity) and sexual attitudes were analyzed by dividing the total score SB into a group with low sexual behavior (respondents with 1–2 points) and a group with high sexual behavior (respondents with 3–4 points) using an independent *t*-test. Also, the differences in sexual behaviors according to general characteristics were analyzed using an independent t-test and one-way ANOVA. For post-hoc analysis, the Scheffe test was conducted. Pearson’s correlation coefficient was used to analyze the correlation between the subcategories of sexual behaviors and attitudes. Multiple linear regression analysis with a stepwise method was performed to examine the influence on the sexual behaviors of college students. The significance level for each statistical value was *p* < 0.05.

## 3. Results

### 3.1. General Characteristics of the Participants

The average age of the participants was 21.38 ± 1.62 (Range: 18~25). Of the participants, 152 (58.9%) were aged ≤21. Furthermore, 214 participants (82.9%) were female, and 62 (24.0%) reported following a religion. The average age of first viewing pornography on YouTube was 14.25 ± 2.55 (Range: 9~22). Moreover, 147 participants (57.0%) responded that they first watched pornography on YouTube at 14 years or younger. Regarding the channels on which they watched pornographic videos, thumbnails and recommendations from friends accounted for 23.3% and 12.8%, respectively. When asked why they watched online pornography, 69.8% said they watched it to relieve stress, and 21.3% responded that it was for sexual curiosity. Last week’s average YouTube viewing time was 2.75 ± 2.41 (Range: 0.1~15) hours (Table 1).

### 3.2. Sexual Behaviors and Sexual Attitudes

The average score converted on a 4-point scale for the sexual behaviors of the participants was 1.86 ± 0.54. The scores for “I once held hands with a friend of the opposite sex” and “I have seen adult pornography (sexual behavior) videos or books” were 2.68 ± 1.03 and 2.62 ± 0.91, respectively, indicating that these two were the most frequent sexual behaviors of the participants. On the contrary, the scores for “I have impregnated or have become pregnant” and “I have been sexually assaulted” were 1.03 ± 0.27 and 1.05 ± 0.23, respectively, indicating that these sexual behaviors are rare among the participants. The group with an SB score ≥ 2 had higher scores for initial sexual activity (1–7 items), advanced sexual activity (8–12 items), and total score of sexual behavior than the group with an SB score < 2, showing significant differences. The mean scores of ‘I have experienced homosexuality’ and ‘I have impregnated or have become pregnant’ were low in both groups, and there was no significant difference between the two groups. The average score converted on a 5-point scale for the sexual attitudes of the participants was 3.05 ± 0.56. The average score of the subcategory of birth control was the highest at 4.71 ± 0.50, followed by communion (3.11 ± 0.83), instrumentality (2.97 ± 0.74), and permissiveness (2.57 ± 0.78). The SB score ≥ 2 groups showed a higher sexual attitude than the SB score < 2 groups in the total score and the subcategories of permissiveness, communion, and instrumentality, showing statistically significant differences. Birth control, a subcategory of sexual attitude, did not show a significant difference between the two groups (Table 2).

### 3.3. Differences in Sexual Behaviors according to General Characteristics

Regarding differences in sexual behaviors according to the general characteristics of the participants, the statistical significance level of sexual behaviors was higher for those aged 21 years or older than for those younger than 21 years (t = −2.38, *p* = 0.018). Females had higher sexual behavior scores than males (t = 2.24, *p* = 0.026). It was also higher for males who first watched online pornography at 14 years or younger compared to those who first watched it at 14 years or older (t = 2.81, *p* = 0.005). Moreover, the statistical significance level of sexual behaviors was higher for those who started watching online pornography based on thumbnails or recommendations by a friend than those who directly searched for and watched online pornography on their own (F = 2.88, *p* = 0.037, Table 3).

### 3.4. Correlations between Sexual Behaviors and the Subcategories of Sexual Attitudes

Sexual behavior was significantly correlated with communion (r = 0.37, *p* < 0.001), instrumentality (r = 0.32, *p* < 0.001), and permissiveness (r = 0.31, *p* < 0.001) (Table 4).

### 3.5. Factors Influencing Sexual Behaviors

To identify the factors influencing the sexual behaviors of college students, the general characteristics of age and the channel through which the participants watched online pornography, the categorical variables showing differences in univariate analysis were set as dummy variables in the analysis. Age and the age at which the participants first watched online pornography were set as continuous variables during hierarchical linear regression analysis. Thus, the researcher developed a regression model for the sexual behaviors of college students (Table 5). When establishing the model, variables were removed at a significant probability of 0.10 and set at a significant probability of 0.05. Model 1 examined the influence of participant characteristics on sexual behaviors. In contrast, Model 2 was designed to examine the influence of the subcategories of sexual attitudes on sexual behaviors when participant characteristics were controlled. In the case of the regression model on the sexual behaviors of college students, the variance inflation factor (VIF) was under 10 at 1.03–2.43, both in Model 1 and Model 2, and the tolerance satisfied the 0.1 criteria at 0.41–0.97, showing that there was no issue of multicollinearity. Additionally, the value of Durbin-Watson was 1.8, and as it was close to 2, the hypothesis of the regression analysis was satisfied.

In Model 1, the influence on the sexual behaviors of participants was higher when they were older, when they first watched online pornography at a younger age, and when they found and watched online pornography through thumbnails. These three variables among the general characteristics were significant influencing factors, and the explanatory power of model 1 was 11.0% (F = 6.27, *p* < 0.001). In Model 2, after controlling for the general characteristic variables of Model 1, communion, instrumentality, and permissiveness, the subcategories of sexual attitudes that showed significant correlation in the correlation analysis were analyzed. The results showed that the higher the level of sexual attitudes in the subcategories of communion and permissiveness, the greater the influence on sexual behaviors. Hence, these two factors were significant influencing factors among the subcategories of sexual attitudes. The explanatory power of model 2 was 24.0% (F = 10.02, *p* < 0.001).

## 4. Discussion

This study was conducted to identify the factors through which the general characteristics and sexual attitudes of Korean college students affect their sexual behaviors. According to the hierarchical regression analysis results in Model 1, sexual behaviors were affected more when the participants were older, when they first watched online pornography at a younger age, and when they watched online pornography after viewing thumbnails. These three variables were found to be significant influencing factors. In Model 2, after controlling for the general characteristic variables of Model 1, the subcategories of sexual attitudes were analyzed. It was found that the higher the sexual attitudes in the subcategories of communion and permissiveness, the more sexual behaviors were affected. The specific details of the influencing factors are as follows.

The results of this study suggest that, among the general characteristics, age plays a significant role, as older participants show a higher level of sexual behavior. This is consistent with the findings of a previous study [19] that older college students are more open-minded toward sexuality. This also aligns with the findings of another study [20], which showed that students from higher grades indicate a higher level of sexual behavior. Among those students, the later they become college students, the more opportunities they have to act concerning their performance, so it is necessary to exercise their sexual autonomy well.

According to the results of this study, those who first watched online pornography at a younger age showed a higher level of sexual behavior. This means that online pornography highly affects the sexual behaviors of college. Previous studies have reported that people who were first exposed to pornography as elementary school students had more liberal sexual attitudes than those who were first exposed to pornography as middle or high school students [21]. It was also stated that people who first watched pornography at a younger age, watch pornography almost every day, or watch pornography for a longer time tend to copy what they saw during sexual intercourse with a person of the opposite sex or tend to be involved in cybersex, as they are more affected by pornography [22]. These results are also consistent with findings suggesting that, as people are more exposed to online pornography, their sexual permissiveness increases [23]. Therefore, appropriate regulations on online pornography need to be introduced, and sex education programs to prevent the abuse of online pornography need to be reinforced.

Specifically, 57% of the participants (more than half) first watched online pornography at 14 years or younger. This is in line with a previous study of college students in South Korea: when they were first exposed to online pornography, 45.7% (36.6 %) were middle school students, 14.3% were high school students, and 3.4% were college students [21]. This indicates that in Korea, students can easily access computers and the Internet, as they are proficient in IT technology, so they are at high risk of being exposed to pornography at an early age. It has been reported that the earlier people start watching online pornography, the earlier their first sexual relationship is, leading to a high possibility of sexual misconduct and active involvement in sexual intercourse [24]. These results suggest that age at first exposure to pornography may have an influence.

Furthermore, the results demonstrate a correlation between individuals who access pornography through thumbnails and their involvement in sexual activities. Given that thumbnails are frequently encountered through the YouTube algorithm, the association between thumbnail exposure and sexual activity suggests that these encounters often happen inadvertently, without a deliberate intention to seek them out. Unlike situations in which people voluntarily and actively search for sexual content, there can be harmful consequences when people are unintentionally exposed to harmful situations because they are in an environment in which such situations naturally exist. Studies conducted in South Korea have reported that some people attempt to commit prostitution or sexual violence after watching pornography [25], and there are concerns about the possibility of the spread of a distorted sexual culture and serious secondary victimization. Moreover, it has been revealed that the more people believe that they can download pornography and sexual content on the Internet at any time, the more likely they are to continue using it [22], showing that exposure to online pornography has serious harmful effects. In the case of YouTube, a commonly used platform for watching videos, the company first introduced machine-learning technology to its existing recommendation algorithm in 2016, offering a completely personalized recommendation service.

Consequently, algorithm-based AI recommendation systems have been widely used [11]. Such an algorithm-based recommendation system could encourage users to watch more pornographic videos, as it consistently recommends related videos once the user is exposed to sexual content. Given these circumstances, where people can easily search for and watch online pornography at any time, we need to encourage sound and healthy sexual behaviors among college students by cultivating their capacity for self-control regarding exposure to online pornography, creating a society culture promoting responsible sexual behaviors, and providing sex education consistently. As college students have reported using the Internet as a source of sexual information [26], it can be concluded that sexual content in the media that can be found online strongly influences college students.

In this study, the level of sexual behavior was higher when the level of sexual attitudes in the subcategories of communion and permissiveness was high. This result is consistent with the findings of previous studies showing that sexual attitude is an influencing factor in sexual behaviors [3] and that those with more open sexual attitudes show more sexual behaviors [27]. A previous study found that college students with permissive sexual attitudes engage in sexual behaviors close to the level of sexual violence [19]. As such, sexual attitude is a factor that affects sexual behavior; therefore, cultivating sound and healthy sexual attitudes is critical. As sexual attitudes affect sexual behaviors [24], it is recommended that college students be educated on the correct sexual knowledge to develop desirable values and knowledge toward sex, which would help them participate in mature sexual behaviors both mentally and physically. Education fosters autonomy by promoting the development of cognitive abilities and ethical frameworks, enabling individuals to make well-informed decisions aligned with their values. Providing sex education on sexual development could help college students adapt to their situations depending on their stage of sexual development and protect them from the risk of their sexual behaviors being triggered [19]. In other words, as sex education influences sexual knowledge, which, once established, affects a person’s values throughout their life and has a powerful impact on sexual behaviors, sex education programs need to be developed for college students. The study revealed both the negative and positive effects of pornography. Negative experiences included inadequacy, decreased sexual satisfaction in relationships, unrealistic expectations, and betrayal. Positive results had increased sexual diversity, greater partner satisfaction, and a more erotic relationship climate [10]. Therefore, it is essential for a neutral and balanced view. They should include content such as what a mature romantic relationship is, the risks of negative sexual behaviors, information on how to control sexual behaviors, and how to prevent sexual violence.

### Limitations

This study was conducted with students from only two universities, so the results should be generalized with caution. Because South Korea has a culture of reluctance to explicitly mention sexual experience, questions about sexual experience can cover a wide range of experiences (e.g., homosexuality, same-sex kissing, sexual intercourse, sexual assault, etc.). Depending on how the students perceived and answered these questions, the results could be slightly different. Therefore, this should be considered when interpreting the results. Furthermore, due to the cross-sectional design of this study, caution is required in interpreting the findings and establishing causal relationships.

## 5. Conclusions

This study found that age, the age at which online pornography was first observed, and sexual attitudes were factors influencing the sexual behaviors of college students. To encourage college students to have sexual autonomy and develop sound sexual behaviors based on positive sexual attitudes, intervention programs should be developed and applied to educate them on what a healthy sexual relationship is and how to enhance the capacity of self-control to reduce the level of exposure to online pornography, including the adverse effects and harmfulness of online pornography. In addition, the government needs to enact the necessary regulations and systems to protect college students from exposure to online pornography. The results of this study are expected to be utilized as useful basic data for developing sex education programs to create a sound sexual culture in our society and for planning policy measures for the issue.

## Figures and Tables

**Table 1:** General Characteristics of the Participants (N = 258).

Characteristics		N	%	M ± SD	Range
Age (year)	21 or less	152	58.9	21.38 ± 1.62	18.00~25.00
	more than 21	106	41.1		
Sex	Female	214	82.9		
	Male	44	17.1		
Religion	No	196	76.0		
	Yes	62	24.0		
Age of first viewing pornography on YouTube (year)	14 or less	147	57.0	14.25 ± 2.55	9.00~22.00
	more than 14	111	43.0		
How to watch pornographic YouTube	Not watching	152	58.9		
	Direct search	13	5.0		
	Thumbnail	60	23.3		
	Recommended by a friend	33	12.8		
Reasons to watch pornographic YouTube	To relieve life stress	180	69.8		
	Sexual curiosity	55	21.3		
	To chat with friends	9	3.5		
	To satisfy sexual desire	14	5.4		
Usual YouTube viewing time (hour/week)	2 or less	151	58.5	2.75 ± 2.41	0.10~15.00
	more than 2	107	41.5		

Notes. M: mean, SD: standard deviation.

**Table 2:** Descriptive Statistics of Sexual Behavior and Sexual Attitudes (N = 258).

Variables	Items	Converted Scores		SB < 2 (n = 121)	SB ≥ 2 (n = 137)	t (*p*)
		M ± SD	Range	M ± SD		
Sexual behavior	12	1.86 ± 0.54	1.0~3.3	1.41 ± 0.19	2.25 ± 0.43	−20.49 (<0.001)
1. I have experience dating the opposite sex.		2.31 ± 0.96	1.0~4.0	1.69 ± 0.52	2.85 ± 0.93	−12.68 (<0.001)
2. I once held hands with a friend of the opposite sex.		2.68 ± 1.03	1.0~4.0	1.93 ± 0.61	3.35 ± 0.85	−15.57 (<0.001)
3. I once kissed a friend of the opposite sex.		2.34 ± 1.19	1.0~4.0	1.45 ± 0.52	3.14 ± 1.04	−16.88 (<0.001)
4. I have done caress.		2.12 ± 1.22	1.0~4.0	1.27 ± 0.45	2.86 ± 1.20	−14.39 (<0.001)
5. I have seen adult pornography (sexual behavior) videos or books.		2.62 ± 0.91	1.0~4.0	2.21 ± 0.52	3.08 ± 0.92	−10.65 (<0.001)
6. I get sexual urges when I see kissing or caressing videos in movies, TV, or magazines.		2.02 ± 0.91	1.0~4.0	1.61 ± 0.58	2.39 ± 0.99	−7.74 (<0.001)
7. I masturbate as a method of relieving sexual desire.		2.10 ± 1.02	1.0~4.0	1.55 ± 0.59	2.59 ± 1.07	−9.80 (<0.001)
Sexual behavior subtotal (1–7 items)	7	2.31 ± 0.77	1.0~3.3	1.66 ± 0.30	2.89 ± 0.58	−21.64 (<0.001)
8. I have experienced homosexuality.		1.09 ± 0.36	1.0~4.0	1.11 ± 0.38	1.08 ± 0.34	0.60 (0.552)
9. I have experience with sexual intercourse.		1.85 ± 1.16	1.0~4.0	1.18 ± 0.47	2.44 ± 1.26	−10.86 (<0.001)
10. I have had sexually transmitted diseases.		1.07 ± 0.36	1.0~4.0	1.00 ± 0.00	1.12 ± 0.49	−2.95 (<0.004)
11. I have impregnated or have become pregnant.		1.03 ± 0.27	1.0~4.0	1.01 ± 0.09	1.04 ± 0.36	−1.11 (0.268)
12. I have been sexually assaulted. (or it has happened.)		1.05 ± 0.23	1.0~4.0	1.02 ± 0.13	1.07 ± 0.29	−2.08 (0.039)
Sexual behavior subtotal (8–12 items)	5	1.22 ± 0.30	1.0~2.8	1.06 ± 0.14	1.35 ± 0.34	−9.25 (<0.001)
Sexual attitudes	23	3.05 ± 0.56	1.7~5.0	2.87 ± 0.46	3.22 ± 0.59	−5.35 (<0.001)
Permissiveness	10	2.57 ± 0.78	1.0~4.9	2.37 ± 0.62	2.75 ± 0.87	−4.11 (<0.001)
Birth control	3	4.71 ± 0.50	2.67~5.0	4.68 ± 0.52	4.73 ± 0.49	−0.75 (0.456)
Communion	4	3.11 ± 0.83	1.0~5.0	2.85 ± 0.78	3.34 ± 0.81	−4.88 (<0.001)
Instrumentality	6	2.97 ± 0.74	1.0~5.0	2.80 ± 0.65	3.12 ± 0.79	−3.67 (<0.001)

Notes. SB: sexual behavior, M: mean, SD: standard deviation.

**Table 3:** Differences in Sexual Behaviors according to General Characteristics (N = 258).

Characteristics		Mean	SD	t or F	*p* (Scheffe)
Age (year)	21 or less	1.78	0.50	−2.38	0.018
	more than 21	1.95	0.58		
Sex	Female	2.02	0.56	2.24	0.026
	Male	18.2	0.54		
Religion	Yes	1.78	0.50	−1.35	0.178
	No	1.88	0.56		
Age of first viewing pornography on YouTube (year)	14 or less	1.93	0.56	2.81	0.005
	more than 14	1.75	0.51		
How to watch pornographic YouTube	Not watching ^a^	1.78	0.51	2.88^*^	0.037
	Direct search ^a^	1.79	0.45		(a < b)
	Thumbnail ^b^	2.00	0.59		
	Recommended by a friend ^b^	1.96	0.59		
Reasons to watch pornographic YouTube	To relieve life stress	1.80	0.53	2.34^*^	0.073
	Sexual curiosity	1.98	0.54		
	To chat with friends	2.11	0.52		
	To satisfy sexual desire	1.95	0.65		
Usual YouTube viewing time (hour/week)	2 or less	1.83	0.55	−0.82	0.415
	more than 2	1.89	0.54		

Notes. ^*^: one-way ANOVA; SD: standard deviation; post-hoc: Scheffe test. Superscripts a and b are subgroups of the Sch1effe test.

**Table 4:** Correlations between Sexual Behavior and Sexual Attitudes (N = 258).

Variables		Sexual Behavior	Sexual Attitudes		
			Birth Control	Communion	Instrumentality
		r (*p*)			
Sexual behavior		1			
Sexual attitudes	Birth control	−0.02 (0.809)	1		
	Communion	0.37 (<0.001)	−0.04 (0.560)	1	
	Instrumentality	0.32 (<0.001)	−0.14 (0.022)	0.49 (<0.001)	
	Permissiveness	0.31 (<0.001)	−0.19 (0.002)	0.33 (<0.001)	0.70 (<0.001)

**Table 5:** Influencing Factors on Sexual Behaviors (N = 258).

Variables	Model 1				Model 2			
	B	SE	t	*p*	B	SE	t	*p*
Intercept	11.78	5.67	2.08	0.039	−0.99	5.67	−0.17	0.862
Age	0.81	0.24	3.30	0.001	0.84	0.23	3.69	<0.001
Age of first viewing pornography on YouTube	−0.55	0.15	−3.54	<0.001	−0.45	0.15	−3.03	0.003
Sex (reference = Male)	1.24	1.05	1.18	0.241	−1.16	1.05	−1.11	0.269
How to watch pornographic YouTube (reference = Not watching)								
Direct search	0.12	1.78	0.07	0.945	0.20	1.65	0.12	0.902
Thumbnail	2.38	0.95	2.52	0.012	1.46	0.89	1.65	0.101
Recommended by a friend	2.19	1.22	1.80	0.073	2.15	1.13	1.90	0.058
Sexual attitudes								
Communion					0.46	0.10	4.43	<0.001
Instrumentality					0.22	0.12	1.88	0.061
Permissiveness					0.15	0.07	2.29	0.023
F (*p*)	6.27 (<0.001)				10.02 (<0.001)			
Adjusted R^2^	0.11				0.24			
Tolerance	0.89~0.97				0.41~0.96			
VIF	1.03~1.13				1.04~2.43			
Durbin-Watson	1.80				1.80			

Notes. B: non-standardized regression coefficient, SE: standard error, VIF: Variance Inflation Factor.

## Data Availability

Data sharing is not applicable.
